# Heterologous overexpression and preliminary antimicrobial activity test of salmocin M, a novel colicin M-like bacteriocin against *Salmonella* sp.

**DOI:** 10.1007/s00203-021-02659-y

**Published:** 2022-01-28

**Authors:** Ewelina Łojewska, Tomasz Sakowicz, Małgorzata Korycka-Machała, Tomasz Kowalczyk

**Affiliations:** 1 Department of Molecular Biotechnology and Genetics, Faculty of Biology and Environmental Protection, University of Lodz, Banacha 12/16, 90-237 Lodz, Poland; 2 Institute of Medical Biology, Polish Academy of Sciences, Lodowa 106, 93-232 Lodz, Poland

**Keywords:** Bacteriocins, *Salmonella*, Bacterial protein production, Antimicrobial activity, Recombinant proteins

## Abstract

Currently, it is extremely important to identify and describe new alternative compounds with potential antimicrobial properties. Since various natural biological systems are capable of producing active compounds with such properties, many of them have been the subject of intensive study. The aim of this work was to heterologously overexpress, purify and preliminarily investigate the antimicrobial activity of a novel bacteriocin found in *Salmonella* species. Overexpressed protein shows an amino acid structure homologous to the well-known colicin M and was never expressed previously in the *E. coli* platform. Purified salmocin M showed an inhibition spectrum against *Salmonella* and *E. coli* strains. To determine its potential as an antimicrobial agent for use in medicine or the food industry, preliminary antimicrobial tests against pathogenic bacteria were carried out. Our research demonstrates that bacteriocin can be produced efficiently in bacterial expression systems, which are one of the cheapest and the most popular platforms for recombinant protein production. Moreover, preliminary results of microbiological tests showed its activity against most of the bacterial strains in a dose-dependent manner.

**Graphical abstract:**

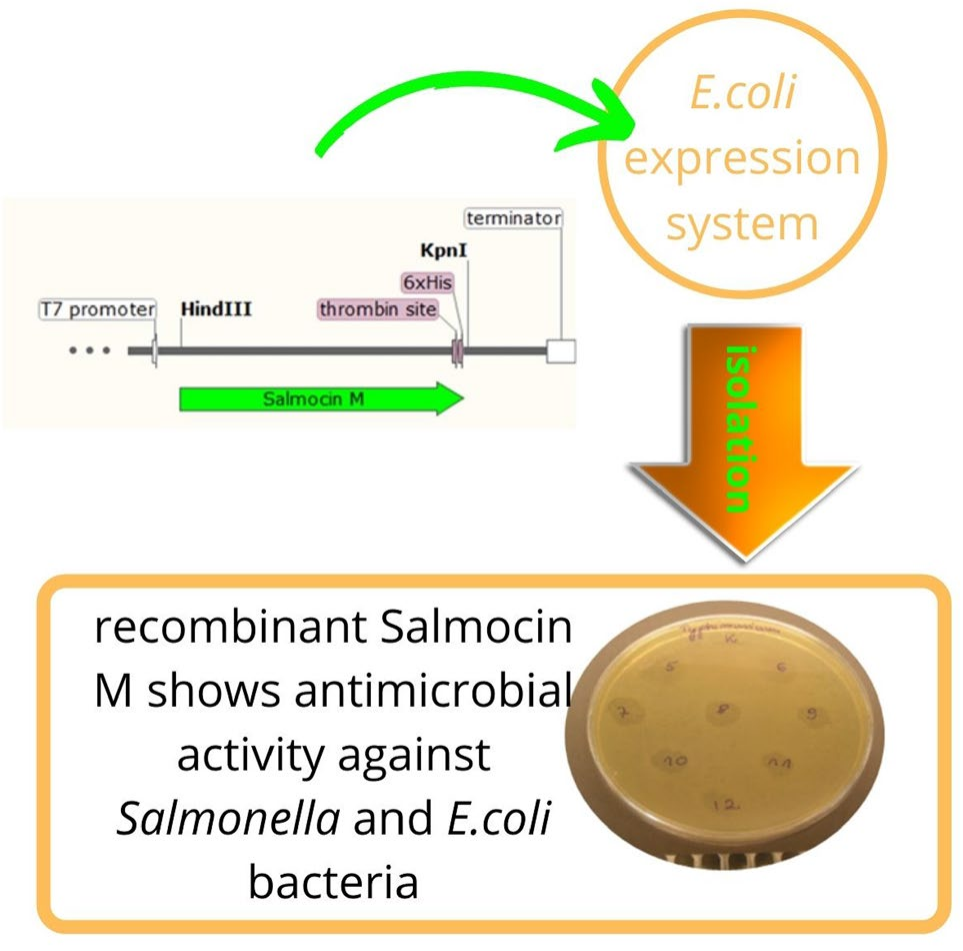

## Introduction

As predicted by experts, bacterial resistance to antibiotics in the coming decades will be a deepening global problem (Laxminarayan et al. 2020). Especially in food production, inappropriate antibiotic usage is linked with the development of resistant bacteria. Measures to counteract this phenomenon are currently taking place on several fronts, including legal restrictions on the use of antibiotics in animal breeding, as well as by searching for alternative antibacterial compounds (Lagadinou et al. 2020). The challenging issue of food safety is still a problem of concern to the scientific community all over the world. The European Commission Action Plan on Antimicrobial Resistance places great emphasis on developing new antimicrobials or alternatives to antibiotics, the European Medicines Agency encourages an increased level of innovation on treatment alternatives for infectious diseases, and a similar situation also takes place in the United States and other countries (Anderson et al. 2020; Mader et al. 2021). Moreover, scientists predict that the COVID-19 pandemic might even increase the rate of bacterial resistance to antibiotics and by 2050 antimicrobial resistance may be responsible for 10 million deaths per year (Strathdee et al. 2020). A large group of microbial infections consists of foodborne illnesses and among them, *Salmonella* is the most common cause of outbreaks in Europe as reported by European Food Safety Authority and European Centre for Disease Prevention and Control (2019). In the United States, *Salmonella* causes an estimated 1.2 million illnesses each year, resulting in an estimated 20,000 hospitalizations and 450 deaths reported by Centre for Disease Prevention and Control (2017). *Salmonella* and *Escherichia coli* gain resistance when exposed to the constant selective pressure of antibiotics, which are increasingly used in the treatment of livestock and then passed on with feces, water, soil, animal products, and even vegetables (Peng et al. 2018; Micallef et al. 2012). Currently, researchers are reporting that *Salmonella* with clinically important resistance caused 29% of outbreaks from land animals and 8% of outbreaks from plant products (Brown et al. 2017). Taking all this into account, there is an urgent need to search for novel antimicrobial agents and build up innovative strategies to combat antibiotic-resistant *Salmonella* causing difficult infections (Nair et al. 2018; Zhang et al. 2018; Nadi et al. 2020).

Nowadays, bacteriocins are one of the most widely described antibacterial alternatives, especially intended for the food industry (Silva et al. 2018; Juturu and Wu 2018; Sang and Blecha 2008; Cotter et al. 2013). Those ribosomal synthesized proteins are used by bacteria to compete against other closely related strains. Bacteriocins have useful features, such as the ability to kill pathogenic bacteria quickly and efficiently. They often act in pico- or nanomolar concentrations. Activity in a wide pH range and tolerance of high thermal stress are other advantages of bacteriocins (Cleveland et al. 2001). Due to their numerous beneficial qualities, they are increasingly used as innovative antimicrobial agents. Moreover, bacteriocins have a narrower spectrum of activity in comparison to antibiotics. They usually only act on a few genera or species closely related to their producer. This fact can be both an advantage and a disadvantage of their potential use. Bacteriocin activity can be very specific, which means that potential therapy would not cause a risk of microbial imbalance as in the case of antibiotic therapy. However, different bacteriocins are needed for the treatment of every group of pathogenic bacteria. Obtaining bacteriocins from strains with potential pathogenicity most often requires the use of genetic engineering tools, which involve a transfer of the bacteriocin-coding gene to a non-pathogenic strain (Arthur et al. 2014). The current results of research on bacteriocins are promising, although further analyses are needed to fully understand their huge diversity, mode of action or cytotoxicity. Therefore, research in this field will be crucial for the continued use of bacteriocins as innovative antibacterial drugs. The colicin group of bacteriocins, produced by *Escherichia coli*, is widely known for its antibacterial and anticancer properties (Kaur & Kaur 2015; Chumchalova et al. 2003). Colicin M is the only bacteriocin that can enzymatically degrade the peptidoglycan lipid II intermediate. Therefore, it is often described as an attractive selective antibacterial agent (Touzé et al. 2012). Colicin M-like bacteriocins were also discovered and described in *Pseudomonas syringae* pv. tomato strain and called syringacin M (Grinter et al. 2012), in *Burkholderia* such homologs were called burkhocins M1 and M2 (Ghequire and De Mot 2015), and the colicin M-like bacteriocin pectocin M2 was identified in *Pectobacterium carotovorum* (Grinter et al. 2014). These proteins are characterized by strong homology of their C-terminus domain to the active antibacterial domain of colicin M. They also have an antibacterial effect similar to colicin M, but extended by activity against a group of closely related bacteria: syringacin M—against *Pseudomonas*, burkhocin M—against strains of *Burkholderia*, pectocin M against *Pectobacterium* strains.

So far very few researchers have addressed the issue of *Salmonella* bacteriocins (Patankar and Joshi 1985). Previous work has only focused on *Salmonella*-derived bacteriocins expressed in plant-based systems and named salmocins, and their activity was also well proven in the literature (Schneider et al. 2018; Hahn-Löbmann et al. 2019). What is more, Generally Recognized As Safe (GRAS) notice for salmocins has recently been published by the FDA (Food and Drug Administration) (also for salmocin M) (GRN 824, https://www.cfsanappsexternal.fda.gov/scripts/fdcc/?set=GRASNotices&id=824&sort=GRN_No&order=DESC&startrow=1&type=basic&search=824). This paper outlines a new approach to salmocin M production, opening a faster path for manufacturing this bacteriocin.

The discovery of new effective molecules that inhibit the growth of zoonotic bacteria can help to replace at least some of the antibiotics currently in use, e.g., in agriculture. However, production systems have to be highly practical and optimized for the scaling-up process. Currently, bacteria are one of the most cost-effective, fast, and easy to scale-up recombinant protein production platforms and that fact is especially important in the case of their potential application and commercialization in agriculture and animal production (Gifre et al. 2017).

## Materials and methods

### Bioinformatic analysis

**Fig. 1:**
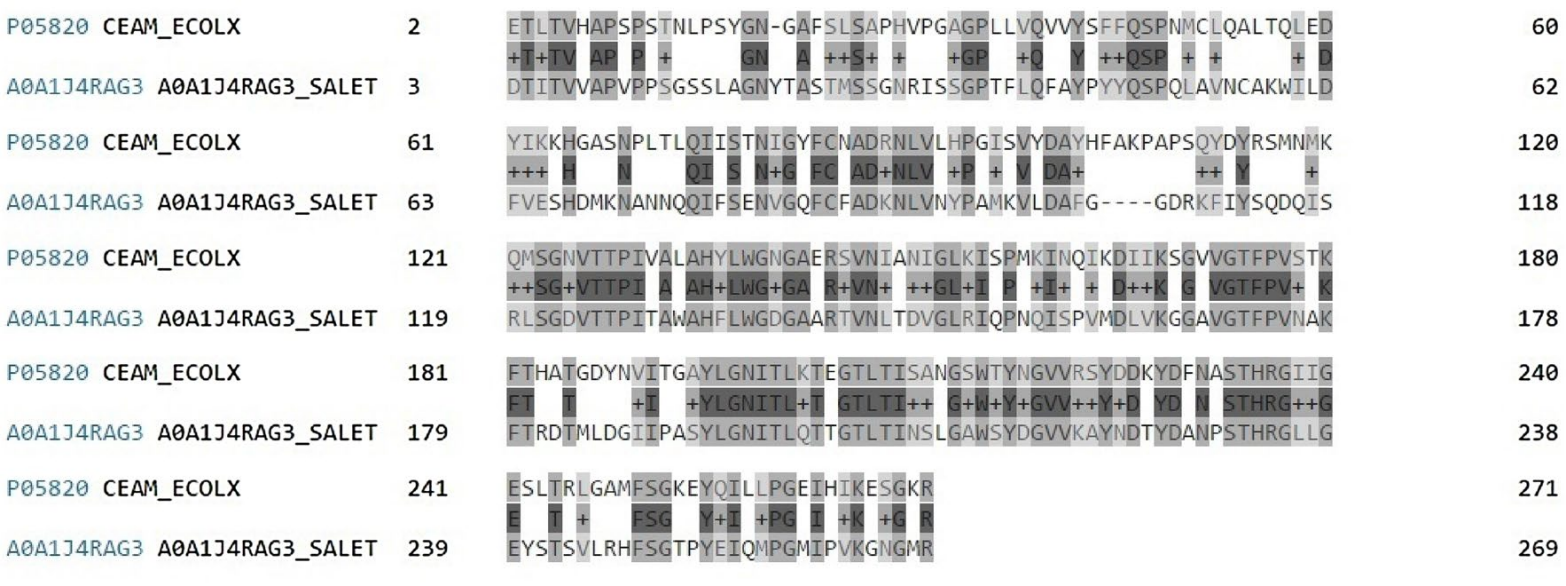
FASTA alignment results of homologic proteins in BLAST—colicin M and the similar protein from *S. enterica I*

The search for colicin M homologs in databases was made using the BLAST program (Fig. 1). These analyses showed the presence of homologs in *Salmonella* genomes. The chosen protein was characterized in BLAST Global Alignment by 44% (119/273) of identities and 64% (177/273) of positives and only 2% of gaps in comparison with colicin M (*cma* gene). It is encoded by the **AO411_2025940** gene of *Salmonella enterica* I (GeneBank accession number: LKLB02000036.1—Table 1). Homology analysis of colicin M and two colicin M-like proteins from *Salmonella* revealed a similarity in the amino acid structure (Fig. 1). The chosen protein amino acid sequence is as follows: MTDTITVVAPVPPSGSSLAGNYTASTMSSGNRISSGPTFLQFAYPYYQSPQLAVNCAKWILDFVESHDMKNANNQQIFSENVGQFCFADKNLVNYPAMKVLDAFGGDRKFIYSQDQISRLSGDVTTPITAWAHFLWGDGAARTVNLTDVGLRIQPNQISPVMDLVKGGAVGTFPVNAKFTRDTMLDGIIPASYLGNITLQTTGTLTINSLGAWSYDGVVKAYNDTYDANPSTHRGLLGEYSTSVLRHFSGTPYEIQMPGMIPVKGNGMRLVPRGSHHHHHH*.

**Table 1:** Protein selected for expression in *E. coli*

Gene	Organism	GeneBank accession number:	Length (amino acids)	Mass (Da)
AO411_2025940	*Salmonella enterica* I subsp. Enterica	LKLB02000036.1	269	29,191

### Construction of expression vector

**Fig. 2:**
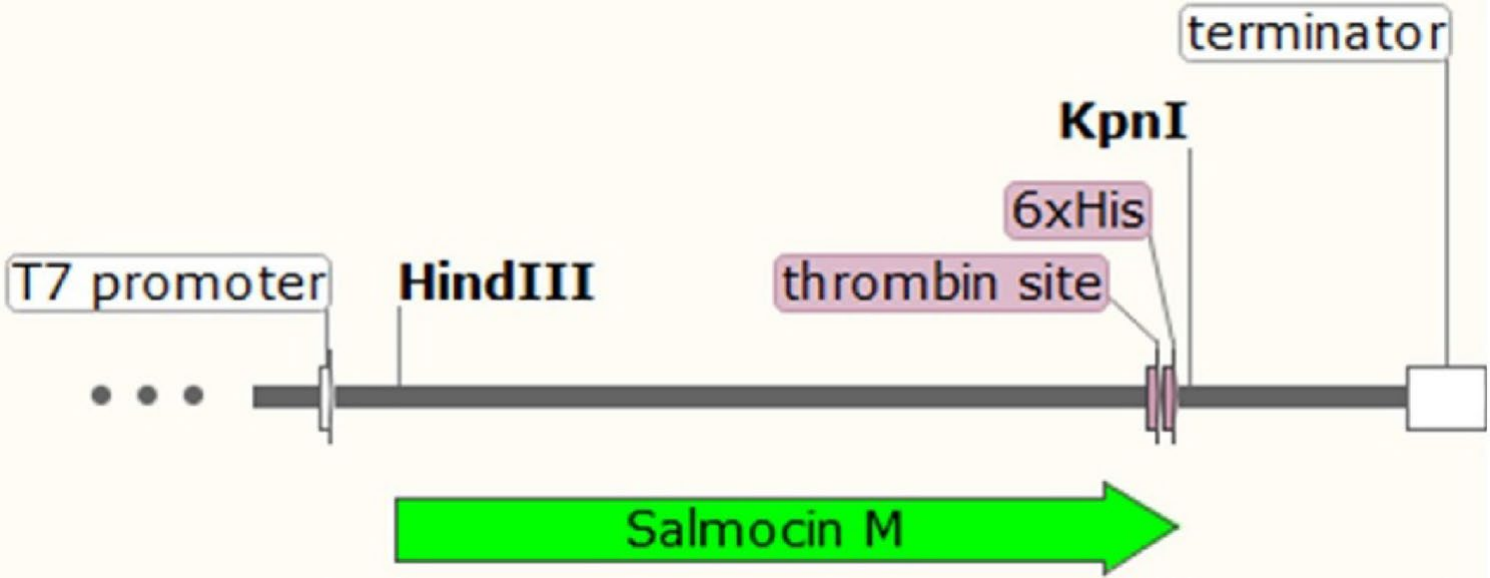
Scheme of the expression cassette used in this work (SnapGene software)

The DNA sequence encoding the tested protein (Table 1) was optimized with the host codon usage pattern for *E. coli* bias (EMBOSS Transeq Sequence Translation) to increase protein yield and then commercially synthesized (Biomatik, Cambridge, Ontario, Canada). Moreover, the addition of His-tag to the C-terminus of protein (with additional thrombin cleavage site) facilitated the purification process by Immobilized Metal Affinity Chromatography (IMAC). The salmocin-coding sequence was introduced into the pT7-MAT-2 expression vector (Sigma-Aldrich) between *HindIII* and *KpnI* restriction sites under the control of a T7 phage promoter (Fig. 2). To confirm the correct construction of the vector, a ligation mixture was used to transform chemically competent *E. coli* DH5-Alpha cells by heat shock (Froger and Hall 2007). Recombinant plasmids were isolated from cells using the Plasmid Mini kit (A&A Biotechnology, Poland), according to the manufacturer’s instructions. The correctness of the recombinant plasmid construction was confirmed by restriction digestion (*Hind*III and *Kpn*I, data not published) and PCR with primers complementary to the AO411_2025940 cloned gene (primer F 5′-ATGAAGCTTATGACCGATACCATTACCG-3′, primer R 5′-TCAGTGATGGTGATGATGATGAGATCCC-3′).

### PCR reaction

PCR was carried out in a Biometra UNOII thermocycler (Biometra, Germany) using a PCR Mix Plus Green (A&A Biotechnology, Poland), by following the instructions provided by the manufacturer. The reaction was carried out in 25 µl and the reaction mixture contained: 12.5 µl PCR Mix Plus Green, 200 nM of F and R primers (2 × 2 µl), 1 µl of template DNA and 7.5 µl of ddH_2_O. The cycling conditions consisted of 4 min of initial denaturation at 95 °C, followed by 30 cycles: 30 s denaturation at 95 °C, 30 s annealing at 55 °C, and a 45 s extension period at 72 °C with a final 5 min extension at 72 °C. The non-recombinant plasmid pT7-MAT-2 was used as a negative control. The amplification products were visualized after electrophoresis on a 1.5% agarose gel and an ethidium bromide staining procedure.

### *E. coli* transformation

The pT7-MAT-2-salmocinM expression vector was used to transform chemically competent *E. coli* strain Rosetta (ƛDE3) cells (Novagen) by the heat shock method. Bacteria were then dispersed on Luria–Bertani (LB) agar plates supplemented with 50 mg/L ampicillin and 34 mg/L chloramphenicol and were incubated overnight at 37 °C. Single colonies were picked and cultured overnight on an LB liquid medium supplemented with 50 mg/L of ampicillin with agitation at 37 °C. The presence of a recombinant plasmid in the transgenic *E. coli* Rosetta cells was finally confirmed by colony PCR using a pair of AO411_2025940 gene-specific primers analogous to PCR performed on an isolated plasmid (data not shown).

### Protein expression and purification

*E. coli* Rosetta transformed with a pT7-MAT-2-salmocinM expression vector were incubated in 5 ml liquid LB (Luria Bertani medium—10 g/L of tryptone, 5 g/L of yeast extract, and 10 g/L of NaCl) for 12 h at 37 °C, 180 rpm in the presence of 50 mg/L ampicillin and 34 mg/L chloramphenicol. 5 flasks of 100 mL TB medium (24 g/L of yeast extract, 20 g/L of tryptone, 4 ml/L of glycerol, 0.017 M KH_2_PO_4_, 0.072 M K_2_HPO_4_) were inoculated with 2 ml of the overnight culture (1:100) and grown at 37 °C, 150 rpm until reaching OD600nm = 0.6. At this point, induction was made by the addition of isopropyl β-d-1 thiogalactopyranoside (IPTG, 0.5 mM) and incubated at 37 °C, 150 rpm for 4 h. After this time, cells were harvested by centrifugation (5000 rpm for 10 min at 4 °C) and lysed by sonication in ice (Amplitude 36%, Bandelin Sonoplus) with the addition of a lysis buffer (50 mM Tris–HCl pH 7.5, 200 mM NaCl) and a 1 mM protease inhibitor—phenyl methyl sulfonyl fluoride (PMSF, Sigma). The soluble fractions of the lysed cells were collected by centrifugation (12,000 rpm for 20 min at 4 °C). The recombinant protein was purified by Immobilized Metal Affinity Chromatography on HIS-Select Nickel Affinity Gel (Sigma-Aldrich). The protein solution was loaded onto an affinity column. The column was washed with a buffer containing 20 mM Imidazole, 20 mM Tris–HCl, 200 mM NaCl. Salmocin M was eluted with 10 ml of an elution buffer containing 150 mM imidazole and finally resuspended in a sterile PBS buffer. The purified recombinant protein was filtered through columns (PD-10 columns Sephadex™ G-25, GE Healthcare) to remove possible bacterial and salt contamination and subsequently it was concentrated via column (VivaspinTM 2 MWCO 30000) up to a concentration of 500 µg/ml. The initial evaluation of the recombinant protein was conducted using SDS-PAGE and compared to the control (proteins of sonicated *E. coli* Rosetta cells without salmocin M expression cassette). SDS-PAGE electrophoresis assessed the effects of purification, and then the protein concentration was evaluated using a modified Bradford method (Pande and Murphy 1994).

### SDS-PAGE

All samples were separated using a 12% SDS-PAGE gel. After that, the proteins were stained with colloidal Coomassie Brilliant Blue G-250 (Dyballa and Metzger 2009).

### Microbiological studies

Antibacterial activity was evaluated on a set of reference *Escherichia coli* strains (DH5-α, NM522) and *Salmonella* strains (*S. *Enteritidis D ATTC13076, *S.* Paratyphi A ATCC 19150, *S.* Typhimurium ATCC 13311,* S.* Gallinarum,* S.* Hadar,* S.* Virchow) spread on a Mueller–Hinton medium agar plate. The recombinant salmocin M used for this study was dissolved in 1 × PBS. Concentrations of the salmocin M tested in a solid medium ranged from 500 to 0.24 µg/ml. The final inoculum of all the organisms studied was 1.5 × 10^8^ CFU/mL (0.5 McFarland standard). Mueller–Hinton agar plates (150 mm) were streaked with a suspension to cover the entire surface of the plates. After the surface of the inoculated plates had dried, 10-µl drops of various concentrations (ranging from 500 to 0.24 μg/ml) of salmocin M were plated on the surface of the agar, and the plate was incubated at 37 °C for 18 h (for the control, a drop (10 µl) of 1 × PBS was used). The diameter of the growth inhibition zone was read after 18 h of incubation at 37 °C. The experiment was performed in 3 repetitions.

## Results

**Fig. 3:**
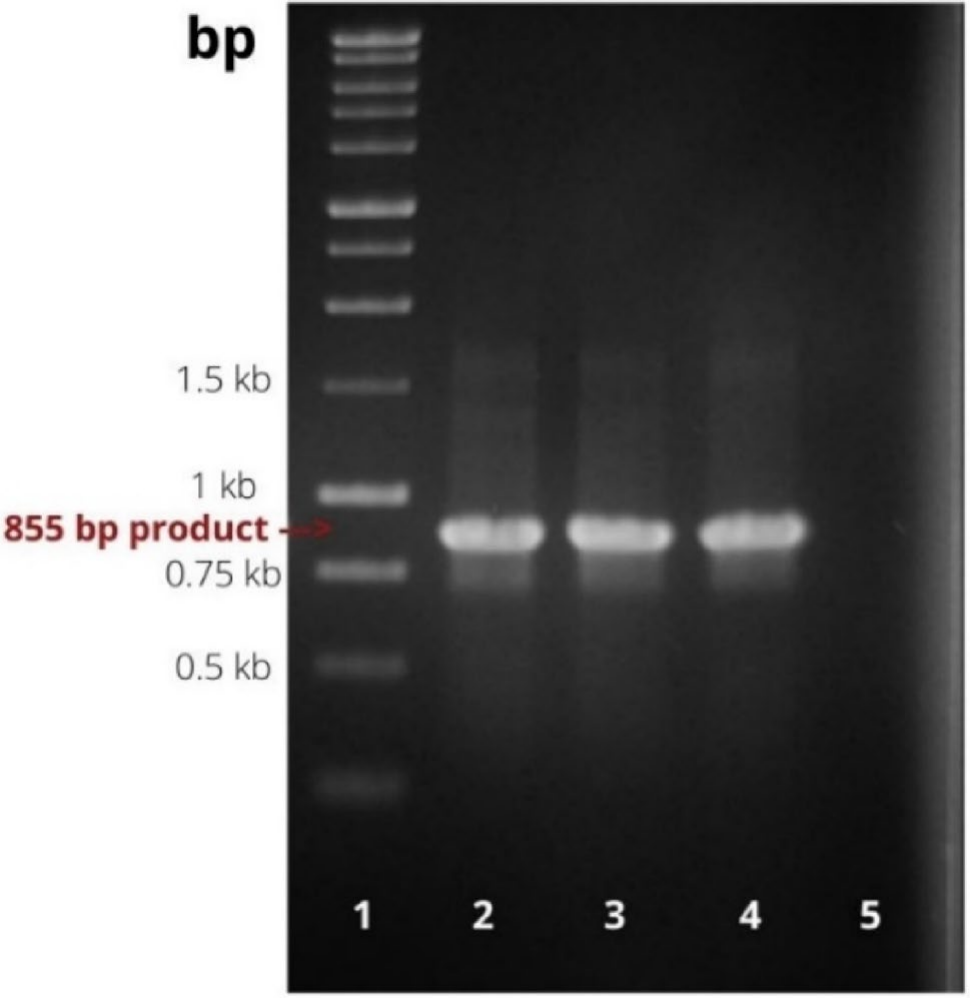
Confirmation of the presence of the insert in the recombinant plasmid. 1—Perfect Plus™ 1 kb DNA Ladder; 2, 3, 4—analyzed DNA samples; 5—negative control with pT7-MAT-2 plasmid

The PCR results confirmed the presence of the insert in the constructed expression cassette. All samples of purified plasmid (and colony PCR, data not shown) that were tested confirmed the presence of the salmocin M gene by the presence of 855 bp amplicons (Fig. 3). In addition, the correctness of the construct was confirmed by restriction hydrolysis using the enzymes* HindIII* and* KpnI*, obtaining DNA fragments consistent with those expected (856 bp and 4784 bp, data not shown).

### Salmocin M expression and purification

**Fig. 4:**
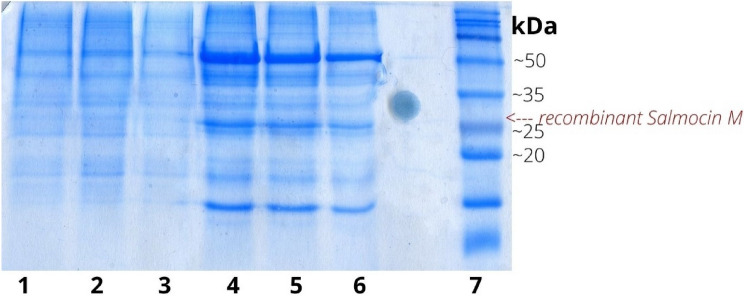
SDS-PAGE electrophoresis of proteins isolated from transgenic and non-transgenic bacterial cells. 1, 2, 3–soluble proteins of non-transgenic *E. coli* cells after 4 h, 3 h, 2 h, respectively from IPTG induction; 4, 5, 6—soluble proteins of transgenic *E. coli* cells after 4 h, 3 h, 2 h, respectively from IPTG induction 7-molecular weight marker. The arrow indicates recombinant protein salmocin M overexpressed in transgenic bacterial cells

Production of recombinant Salmocin M has been observed in transformed *E. coli,* unlike untransformed cells*.* In all samples, SDS-PAGE analysis indicated the presence of an approximately 30 kDa band in the soluble protein samples (Fig. 4).

**Fig. 5:**
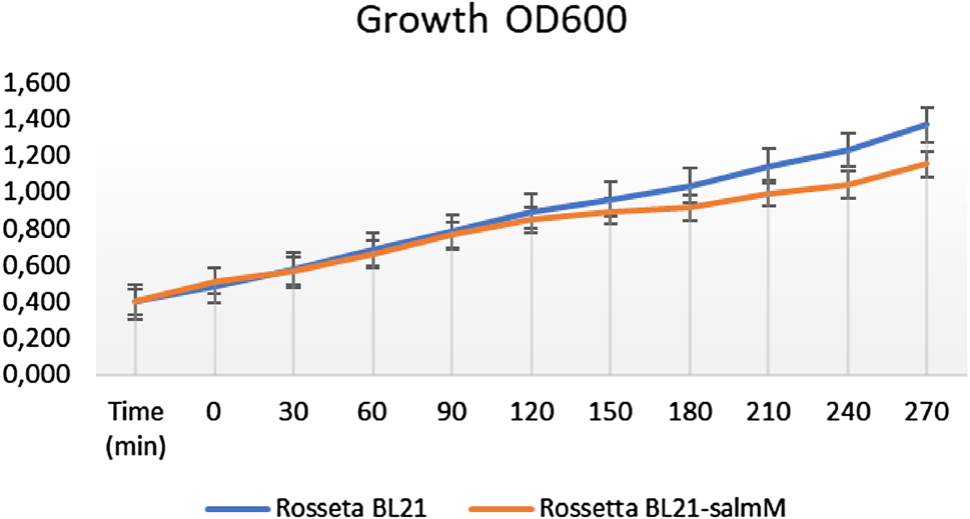
Time dependence of bacterial growth at OD600 starting from IPTG induction at OD600 = 0.4

The maximal expression yield was obtained at 37 °C for 4 h after the induction (Fig. 4) without significantly affecting bacterial growth (Fig. 5). Therefore, these conditions were used for protein production. Almost all recombinant salmocin M appeared in the soluble fraction. Out of the 500 ml liquid culture of *E. coli* expressing pT7-MAT-2-salmocin M, 0.25 mg of salmocin M was obtained.

**Fig. 6.**
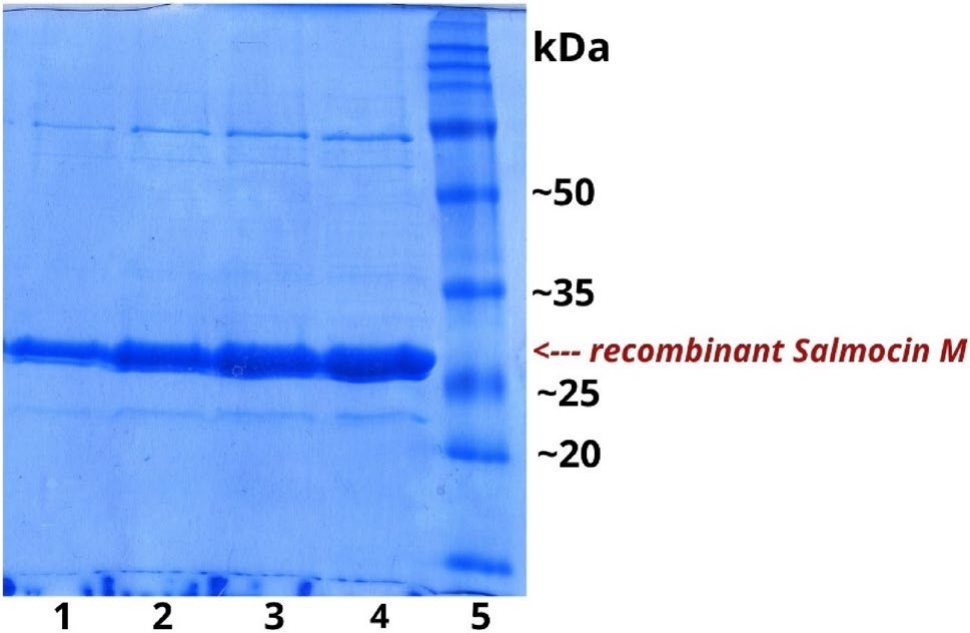
Four stages of purified protein concentration on SDS-PAGE gel (12%)

Our results showed that the applied recombinant protein purification strategy was effective as evidenced by the SDS-PAGE of purified protein samples (Fig. 6).

### Antimicrobial activity screening

**Fig. 7:**
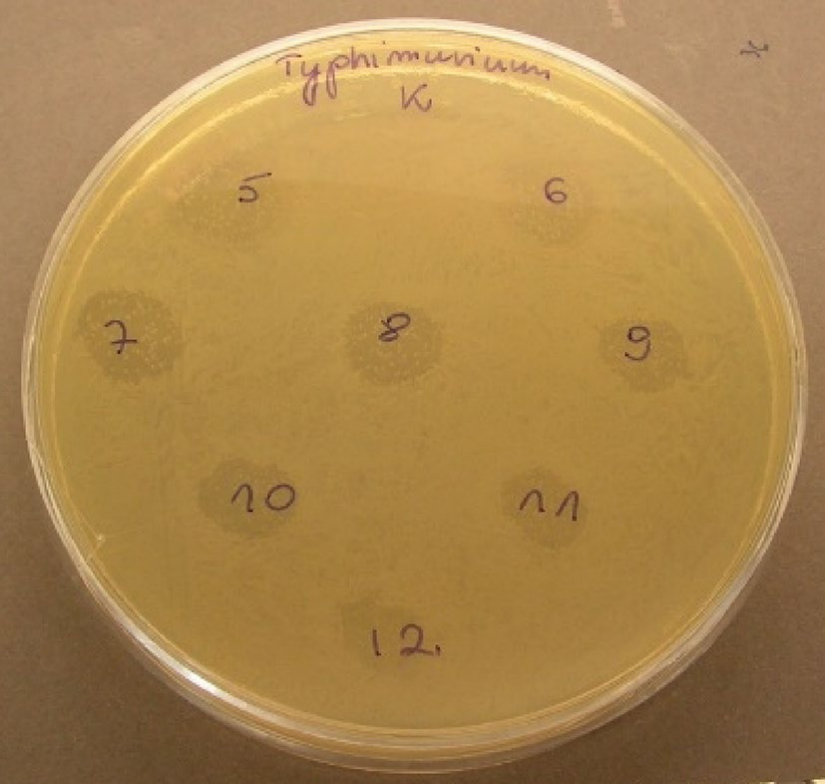
The activity of salmocin M in different concentrations

**Fig. 8:**
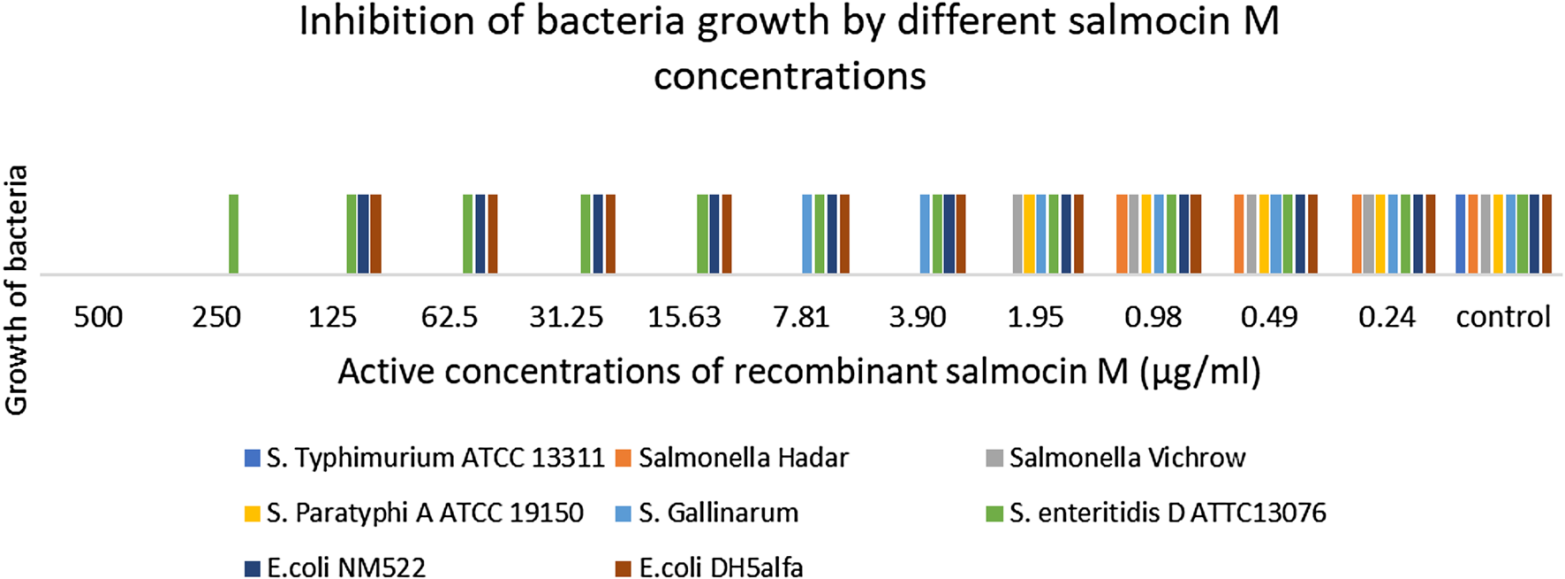
Inhibition of *Salmonella *Typhimurium growth zones by different concentrations of salmocin M (K-control sterile PBS; 5—31.25 µg/ml of protein; 6—15.63 µg/ml; 7—7.81 µg/ml; 8—3.90 µg/ml; 9—1.95 µg/ml; 10—0.98 μg/ml; 11—0.49 μg/ml; 12—0.24 μg/ml

Purified recombinant salmocin M was obtained in its active form. The protein proved to have antimicrobial activity on all bacteria tested, as is illustrated in Fig. 7 Bacteriocin activity was concentration dependent although for two *Escherichia coli* the minimal active concentration was 250 µg/ml, while the majority of *Salmonell*a strains were more susceptible to the protein studied. Table 2 summarizes the data on the concentration ranges that lead to the growth inhibition of bacteria on the plate. The recombinant protein was active in the concentration starting from 0.24 µg/ml for *Salmonella* Typhimurium*,* which was the most susceptible strain of all those tested, as shown in Fig. 8. Generally, *Salmonella* strains are more susceptible to low concentrations than the *Escherichia coli* strains tested, which is a promising result.

**Table 2:** The activity of salmocin M in different concentrations

Bacteria strain	Active concentrations of recombinant salmocin M (µg/ml)												
	500	250	125	62.5	31.25	15.63	7.81	3.90	1.95	0.98	0.49	0.24	0-control
*S. *Typhimurium ATCC 13311	A	A	A	A	A	A	A	A	A	A	A	A	N
*Salmonella* Hadar	A	A	A	A	A	A	A	A	A	n	n	n	N
*Salmonella* Vichrow	A	A	A	A	A	A	A	A	n	n	n	n	N
*S.* Paratyphi A ATCC 19150	A	A	A	A	A	A	A	A	n	n	n	n	N
*S.* Gallinarum	A	A	A	A	A	A	n	n	n	n	n	n	N
*S. *Enteritidis D ATTC13076	A	n	n	n	n	n	n	n	n	n	n	n	N
*E. coli* NM522	A	A	n	n	n	n	n	n	n	n	n	n	N
*E. coli* DH5alpha	A	A	n	n	n	n	n	n	n	n	n	n	N

## Discussion

Colicin M-like bacteriocins have gained considerable interest for their potential use as food preservatives in recent years. Given the great need to obtain new compounds with antimicrobial activity, many studies are currently being conducted to identify and use in practice such compounds from natural sources. Due to their medical and preservative properties, bacteriocins are being isolated from different organisms. However, the majority of them is derived from lactic acid bacteria (Mokoena 2017; da Costa et al. 2019; Barbosa et al. 2017; Field et al. 2018), and according to research, they can act even as growth promoters (Vieco-Saiz et al. 2019). However, different species are also considered interesting sources, for example, *Bacillus* (Abriouel et al. 2011), *Enterococcus* (Chang et al. 2013), *Pediococcus* (Anastasiadou et al. 2008), *Gluconacetobacter* (Oliveira et al. 2018), *Carnobacteria* (Acedo et al. 2017) and other genera (Masuda et al. 2011; Masuda et al. 2012; Marques-Bastos et al. 2020). The possibility of using advanced genetic engineering tools allows the application of various expression systems to avoid virulence factors of hosts. In our previous work, we demonstrated the possibility of colicin M biosynthesis in transgenic plants (Łojewska et al. 2020). However, due to well-developed and fast manipulation methods, *E. coli* can act also as an economic and efficient host for bacteriocin production with a long history of such studies (Richard et al. 2004; Tang et al. 2018; Tapia et al. 2011; Chen et al. 2012). In this study, the structural gene for salmocin M was identified from *Salmonella enterica I*, cloned into an expression vector for *E. coli*, overexpression of this recombinant protein was obtained, and then the isolated and purified protein was used for preliminary evaluation of antibacterial activity against *E. coli* and *Salmonella*. In this case, due to the speed of obtaining proteins for our preliminary antimicrobial research, as well as the high efficiency of the bacterial system, we decided to produce salmocin M in a non-pathogenic *Escherichia coli* Rossetta (ƛDE3) host. In this work, for the first time, salmocin M was overexpressed and purified successfully in *E. coli* cells. Our preliminary results confirmed the successful pT7-MAT-2 expression vector recombination resulting in the construction of an expression cassette containing the salmocin M gene under the control of the T7 promoter. Both PCR and SDS-PAGE analysis confirmed the presence of the gene in the recombinant vector and the expression of the recombinant protein in *E. coli* cells, respectively. Generally, it is sometimes difficult to produce antimicrobial recombinant proteins in prokaryotic expression platforms. Such proteins are potentially susceptible to proteolytic degradation and, most importantly, toxic to the host cells. However, those inconveniences can be avoided by using different techniques of optimization, starting from the expressed protein (e.g. addition of tags and/or other fusion proteins) to selecting specific conditions of culture and cell harvesting or expression induction strategy (Saida et al. 2006; Wanmakok et al. 2018). In line with the selected recommendations of many authors cited in this work, we shortened the cultivation time after induction by IPTG to obtain the optimal yield of undegraded recombinant protein. Additionally, purification of bacteriocin was achieved with Immobilized Metal Affinity Chromatography on HIS-Select Nickel Affinity Gel using a His-tag. Our preliminary data showed that protein retained its antibacterial activity despite the presence of His-tag and thrombin cleavage site allowing for His-tag removal after the purification. Our results are consistent with those of Ghachi et al., who showed that the attachment of a His-tag to colicin M did not affect its activity (El Ghachi et al. 2006).

Due to the fact that the aim of this study was the expression and only preliminary examination of the antibacterial properties of the identified protein, the protein production efficiency in this system was not optimized in detail.

The results of our preliminary antibacterial studies showed that the protein being tested was active against *S.* Virchow, *S.* Hadar,* S.* Typhimurium and *S. *Enteritidis, which are the main cause of salmonellosis, one of the most common food poisoning, possibly leading to sepsis and death. Moreover, recombinant protein was also active against *S. *Paratyphi, bacteria causing paratyphoid fever, a disease currently cured with antibiotics, although *S.* Paratyphi becomes increasingly resistant to them. Salmocin M had the highest effectiveness against *Salmonella* strains, among which the strongest effect was obtained for *S.* Typhimurium (0.24 µg/ml) and the weakest for *S.* Enteritidis D ATTC13076 (500 µg/ml). Among the two *E. coli* strains tested, recombinant salmocin M showed a similar activity at the level of 250 µg/ml. Hahn-Löbmann et al. (2019) also showed that the tested salmocins SalE1a and SalE1b revealed the strongest effect on *S.* Typhimurium. Contrary to our results, the salmocins studied by these authors also showed strong activity against *S.* Enteritidis.

Bacteriocins are natural, non-antibiotic proteins with antibacterial properties with an extremely wide potential application from medicine to the broadly understood industry. The possibility of using them to reduce or eliminate pathogenic bacteria opens up the possibility of enriching the antimicrobial arsenal alternative to commonly used and often overused antibiotics.

These results allow the assumptions about the antibacterial activity of *Salmonella* bacteriocins to be confirmed, but further research is necessary to investigate the mechanisms of its action, which in the future may allow for the development of a new effective alternative to combat these pathogenic bacteria.
